# Supplementary material for: Enhanced language models for predicting and understanding HIV care disengagement: a case study in Tanzania
Source: NPJ Digit Med. 2026 Jan 21;9:165. doi: 10.1038/s41746-026-02349-3 (PMC12909982; doi:10.1038/s41746-026-02349-3)
Supplement: Supplementary file 1 — Supplementary information [file 41746_2026_2349_MOESM1_ESM.pdf]

Supplementary Material

LLM prompt input: Summary	LLM Prediction	True Label	LLM Reasoning
The patient, a 48-year-old male living with HIV, has a history of missed or delayed appointments, with gaps in follow-up care noted in 2020 and 2021. The patient missed appointments on 2020-04-16, 2021-03-11, and 2021-09-13, with delays of 2, 2, and 4 days in ART refills, respectively. The number of pills dispensed varied over time, with an increase from 30 to 90 pills in 2020 and a subsequent increase to 180 pills in 2021. There were fluctuations in weight, with a decrease from 65 kg to 61 kg in 2020, followed by an increase to 64 kg in 2021. The patient's engagement with care improved over time, transitioning from an unstable client to a stable client category. The patient consistently continued ARV treatment, with suppressed viral load noted in some visits.	The patient is at [no] risk of disengaging from HIV care, has a [suppressed] viral load, and is [unlikely] to be lost to follow up for over 28 days in year 2022-2023.	The patient is at [no] risk of disengaging from HIV care, has a [suppressed] viral load, and is [unlikely] to be lost to follow up for over 28 days in year 2022-2023.	The patient had a history of consistent appointment adherence, had a suppressed viral load, and had a strong support system in place. Furthermore, the patient has been actively engaged in their HIV care, has had a high level of health literacy, and has had a strong relationship with their healthcare provider.

Supplementary Table 1. A synthetic example (to protect individual privacy) of summarized textual medical record-based LLM prompt input, prediction, true label, and reasoning.

	**Internal** validation in the Kagera Region			**External** validation in the Geita Region		
Subgroups	Risk category	Viral suppression	LTFU	Risk category	Viral suppression	LTFU
Enhanced LLM with **summarized** CTC3 medical records						
2021 not-in-care	0.82	0.66	0.89	0.69	0.63	0.82
2021 in-care	0.67	0.62	0.70	0.61	0.60	0.65
Age < 26	0.60	0.60	0.63	0.58	0.60	0.62
26 < Age < 42	0.68	0.65	0.70	0.62	0.62	0.66
Age > 42	0.77	0.73	0.81	0.68	0.65	0.71
Male	0.74	0.65	0.83	0.67	0.62	0.70
Enhanced LLM with summarized and raw CTC3 medical records						
2021 not-in-care	0.82	0.67	0.89	0.68	0.63	0.78
2021 in-care	0.69	0.64	0.70	0.64	0.60	0.67
Age < 26	0.60	0.61	0.66	0.60	0.60	0.64
26 < Age < 42	0.69	0.67	0.71	0.65	0.64	0.7
Age > 42	0.78	0.74	0.83	0.71	0.69	0.8
Male	0.75	0.66	0.84	0.67	0.66	0.77
Supervised machine learning (gradient boosting)						
2021 not-in-care	0.62	0.68	0.53	0.58	0.65	0.56
2021 in-care	0.71	0.67	0.73	0.63	0.61	0.64
Age < 26	0.66	0.65	0.65	0.61	0.60	0.60
26 < Age < 42	0.71	0.67	0.71	0.65	0.64	0.66
Age > 42	0.71	0.67	0.75	0.63	0.64	0.70
Male	0.72	0.71	0.73	0.66	0.66	0.69
Zero-shot LLM with summarized CTC3 medical records						
2021 not-in-care	0.48	0.48	0.49	0.47	0.46	0.47
2021 in-care	0.52	0.50	0.52	0.51	0.51	0.52
Age < 26	0.58	0.55	0.61	0.57	0.53	0.6
26 < Age < 42	0.48	0.47	0.51	0.49	0.48	0.52
Age > 42	0.45	0.47	0.45	0.46	0.48	0.47
Male	0.49	0.48	0.5	0.51	0.49	0.51
Zero-shot LLM with summarized and raw CTC3 medical records						
2021 not-in-care	0.50	0.50	0.51	0.46	0.45	0.47
2021 in-care	0.50	0.51	0.53	0.50	0.50	0.54
Age < 26	0.61	0.60	0.63	0.58	0.53	0.61
26 < Age < 42	0.50	0.51	0.51	0.48	0.50	0.51
Age > 42	0.47	0.46	0.47	0.47	0.47	0.48
Male	0.50	0.50	0.53	0.51	0.50	0.54

Supplementary Table 2. Subgroup analysis of enhanced LLM model performance (ROC-AUC) on the internal and external validation datasets.

	Training data	Internal validation	External validation	Combined two regions
	Kagera Region (80%)	Kagera Region (20%)	Geita Region	
**Sample size: patients**	51575 patients	12894 patients	38933 patients	103402 patients
**Sample size: records**	965127 records	242063 records	647030 records	1854220 records
**Age in years (Median, IQR)**	33.0 (16.0)	33.0(15.0)	33.0 (16.0)	33.0 (15)
**Weight (Median, IQR)**	56.0 (12.0)	56.0 (13.0)	56.0 (12.0)	56.0 (12.0)
**Time from HIV diagnosis to ART start, days (Median, IQR)**	1007.0 (1781.0)	1004.0 (1769.0)	762.0 (1373.0)	911.0 (1624.0)
**Number of Days Dispensed (Median, IQR)**	30.0 (60.0)	30.0 (60.0)	30.0 (30.0)	30.0 (60.0)
**Time on ART, days (Median, IQR)**	969.0 (1525.0)	972.0 (1519.0)	777.0 (1351.0)	898.0 (1473.0)
**Sex (Counts by patients, %)**				
Male	19522(37.9%)	4968(38.5%)	14472 (37.2%)	38962 (37.7%)
Female	32053(62.1%)	7926(61.5%)	24461 (62.8%)	64440 (62.3%)
**Referral (Counts by records, %)**				
1	383453 (39.7%)	94057 (38.9%)	313078 (48.4%)	790588 (42.6%)
2	183194 (19.0%)	48560 (20.1%)	92445 (14.3%)	300253 (16.2%)
3	119066 (12.3%)	29283 (12.1%)	76195 (11.8%)	240794 (13.0%)
4	114397 (11.9%)	28754 (11.9%)	70154 (10.8%)	214592 (11.6%)
5	110316 (11.4%)	28081 (11.6%)	68499 (10.6%)	213305 (11.5%)
0	54701 (5.7%)	13328 (5.5%)	26659 (4.1%)	94688 (5.1%)
**Marital Status (Counts by record, %)**				
M	539739 (55.9%)	135673 (56.0%)	350194 (54.1%)	1025606 (55.3%)
S	135150 (14.0%)	33653 (13.9%)	88094 (13.6%)	256897 (13.9%)
D	103128 (10.7%)	24670 (10.2%)	66739 (10.3%)	194537 (10.5%)
W	52137 (5.4%)	13324 (5.5%)	25035 (3.9%)	90496 (4.9%)
C	10813 (1.1%)	2626 (1.1%)	17426 (2.7%)	30865 (1.7%)
CO	8582 (0.9%)	2240 (0.9%)	5656 (0.9%)	16478 (0.9%)
Missing	114501 (11.9%)	29506 (12.2%)	92760 (14.3%)	236767 (12.8%)
**Health facility type (Counts by record, %)**				
1	458295 (47.5%)	113456 (46.9%)	313988 (48.5%)	885739 (47.8%)
2	288092 (29.9%)	72364 (29.9%)	294286 (45.5%)	654742 (35.3%)
3	218740 (22.7%)	56243 (23.2%)	38756 (6.0%)	313739 (16.9%)
**WHO stage (Counts by record, %)**				
1	417142 (43.2%)	103393 (42.7%)	219146 (33.9%)	739681 (39.9%)
2	223652 (23.2%)	55429 (22.9%)	128633 (19.9%)	336032 (18.1%)
3	165211 (17.1%)	42188 (17.4%)	173423 (26.8%)	452504 (24.4%)

4	71134 (7.4%)	18613 (7.7%)	55808 (8.6%)	145555 (7.8%)
Missing	87988 (9.1%)	22440 (9.3%)	70020 (10.8%)	180448 (9.7%)

Supplementary Table 3. Dataset Description: Characteristics of PLHIV since Jan 2018 from Geita and Kagera regions in CTC3 medical records.

**Variable Type**	**Variable Name**	**Description**
Structural	txcurr	Current treatment (NA, 1)
Structural	ID	Patient ID
Structural	date	Patient visit date
Structural	VisitDate	Patient visit date
Structural	visitdate	Patient visit date
Structural	DateAppointmentGiven	Given appointment data
Structural	StatusDate	Patient status date
Structural	TestDate	Patient testing date
Structural	FacilityName	Facility name (372 different facilities in total)
Structural	facility_name6	Facility name (373 different facilities in total)
Structural	facility_name	Facility name (372 different facilities in total)
Structural	hf_type	Health facility type 1: Dispensary; 2: Health Center; 3: Hospital
Structural	Region	Region (Kagera or Geita)
Structural	Council	District Council
Structural	DateofBirth	Patient date of birth
Structural	Sex	Patient Sex

Structural	DateofDeath	Patient date of death
Structural	DateFirstPositiveHIVTest	Date of patients’ first positive HIV test
Structural	DateConfirmedHIVPositive	Date of patients’ confirmed positive HIV
Structural	PriorExposure	Patient's antiretroviral treatment (ART) or prophylaxis history before initiating care at the current facility
Structural	MaritalStatus	Patient marital status<br>C/HH = Child (<15 years)/Haihusiki (Not applicable)<br>M = Married<br>CO = Cohabitating<br>D/WT= Divorced/Separated/Wametengana (Separated)<br>S = Single<br>W = Widow/widowed<br>NW = Ndoa wake wengi (Polygamy)
Structural	DrugAllergies	Patient drug allergies
Structural	WardName	Administrative ward in which the patient received care or is registered (239 different wards in total)
Structural	VillageMtaa	Smallest administrative unit where the patient resides or receives services (335 different villages or mtaas in total)
Free text	ReferredFromID	Souces from which patients were referred to the clinics
Structural	ref_final_new	Facilities where the patients were referred to
Structural	ref_final	Final referral source or method<br>0:NULL;1: PITC;2: VCT; 3:INDEX; 4:COMMUNITY; 5:OTHER
Structural	other	Other or unknown source<br>0: No<br>1: Yes<br>NA
Structural	indexfac	Index testing at facility level<br>0: No<br>1: Yes<br>NA
Structural	cbhs	Community-Based HIV Services<br>0: No<br>1: Yes<br>NA
Structural	pmtct	Prevention of Mother-To-Child Transmission services<br>0: No<br>1: Yes<br>NA

Structural	vmmc	Voluntary Medical Male Circumcision program testing (0, NA, 1, 6, 5, 11, 2)
Structural	transfer	Transferred from another facility<br>0: No<br>1: Yes<br>NA
Structural	pitc	Provider-Initiated Testing and Counseling<br>0: No<br>1: Yes<br>NA
Structural	opd	Outpatient Department<br>0: No<br>1: Yes<br>NA
Structural	vct	Voluntary Counseling and Testing<br>0: No<br>1: Yes<br>NA
Structural	self	Self-referred or self-testing<br>0: No<br>1: Yes<br>NA
Structural	ipd	Inpatient Department (0, 1, NA, 9, 5)
Structural	CommunitySupportOrg	Community-based support organization (“Null, “”)
Structural	TheHeight_num	Patient height (numeric)
Structural	TransferInID	Type of patient transfer<br>ARNR: Another facility, Referral, Not Registered<br>ARRC: Another facility, Referral, Registered Client<br>CRRC: Community-based, Referral, Registered Client<br>CRNR: Community-based, Referral, Not Registered<br>NULL: No transfer-in information provided (patient was not transferred).
Structural	maxheight	Patient maximum height during visits
Structural	VisitTypeCode	Patient visit type code:<br>US: Unscheduled visit at this clinic; S: Scheduled visit at this clinic; TK: Traced back after LTFU; RV: Refill visit; TS: Treatment supporter drug pick up; O: Visit other clinic; Rvo: Refill visit - facility-led - outreach community refill; RVp: Refill visit - fast track refill at health facility pharmacy; IP: In-patient consultation
Structural	Weight	Patient weight
Structural	TheHeight	Patient height (character)

Structural	WHOStage	WHO Stage 1 (1) = 1<br>WHO Stage 2 (2) = 2<br>WHO Stage 3 (3) = 3<br>WHO Stage 4 (4) = 4
Structural	NowPregnant	Patient pregnancy status
Structural	FamilyPlanningID	Family planning methods<br>A: Abstaining<br>C: Condom<br>N: Pregnant<br>R: Cannot be found as of now
	TBScreeningID	TB screening ID
Structural	TBRXIPTID	NO IPT = Eligible but not started TPT<br>START 3HP = START 3HP<br>START INH = START INH Prophylaxis<br>START IPT = START IPT<br>CTN IPT = CONTINUE TPT<br>STOP IPT = STOPPED TPT<br>RES INH = RESTART INH<br>RES IPT = RESTART INH<br>CPLT TPT = COMPLETE TPT
Structural	tb_tpt	0:Not on TB preventive therapy (TPT);1: Start taking TPT; 2: Continue taking TPT;3: Complete taking TPT; 4: Stop taking TPT; 5: Restart taking TPT
Structural	AntiTBStartDate	Start date of anti-TB treatment
Structural	NoDaysIPTDrugsDispensed	Number of days isoniazid preventive therapy (TB prevention) prescribed
Structural	ARVStatusCode	ARV status code<br>1: No ARV<br>2: Start ARV<br>3: Continue ARV<br>4: Change ARV<br>5: Stop ARV<br>6: Restart ARV<br>8: ARV substitution<br>9: Switch to 2nd line<br>10: Switch to 3rd line
Structural	ARVCode	ARV combination regimen (62 distinct values)
Structural	ARVDescription	Description of ARV
Structural	V2Code	Visit classification code (61 distinct values)
Structural	RegimenType	Regimen type: First line, Second line, Third line, Other, Prophylaxis
Structural	NumDaysDispensed	Number of days dispensed pills

Structural	ARVAdherenceCode	ARV adherence code:<br>G: good, >=95% adherence<br>P: poor, <=95% adherence
Structural	ClientCategory	Client category<br>UN: Unstable client<br>S: Stable client<br>EP: Early presentation<br>LP: Late presentation<br>LPA: Late presentation with advanced disease (CD4<200)
Structural	NutritionalStatusID	Nutritional status ID<br>OK: Not malnourished<br>MOD: Moderately malnourished<br>SEV: Severely malnourished<br>OB: Obesity
Structural	Cancelled	Appointment cancelled (0, Null, -1)
Structural	DateOfAppointment	Appointment date
Structural	Status	Patient status: Attending this clinic, Missing appointments, Lost to follow-up, Transferred to another clinic, Died, Opted out, Confirmed HIV positive, Not HIV positive
Structural	TestTypeID	Test type:<br>VRL: viral load<br>CD4: CD4 count<br>HBX: Hemoglobin test<br>CD%: CD4 percentage<br>ALT: Alanine Aminotransferase test<br>WBC: white blood cell count<br>OTH: other tests<br>CRT: Creatinine test<br>TL%: Total lymphocyte percentage<br>TLC: Total lymphocyte count
Structural	ResultDate	Date of test results
Structural	ResultNumeric	Numerical values of test results
Free text	ResultNotes	Notes on result
Structural	ResultReturnDate	Date of test results returned
Structural	DateInitiatedEAC	Date of EAC initiation
Structural	DateHVLTestAfterEAC	Date of HVL test after EAC
Structural	missing	Missing visits

Structural	merge1	Data merging operation (3, NA)
Structural	merge2	Data merging operation (1, 2, 3, NA)
Structural	merge3	Data merging operation (1, 2, 3, NA)
Structural	merge4	Data merging operation (1, 2, 3, NA)
Structural	dup_details	Number of duplicate records or entries
Structural	dup_visitdate	Number of duplicate records or entries on the visit date
Structural	dup1	Number of duplicate records or entries
Structural	dup_appointment	Number of duplicate records or entries on the appointment
Structural	dup2	Number of duplicate records or entries
Structural	dup_status	Number of duplicate records or entries on the status
Structural	dup_test	Number of duplicate records or entries on tests
Structural	fullfacilitycode	Location facility code
Structural	locationfacilitycode	Location facility code
Structural	hf_own	Health facility ownership<br>1:FBO; 2:Private; 3:Public
Structural	tag	Tag indicator (1, NA)
Structural	datereadystartart	Date ready to start ART
Structural	ref_final_original	NULL; PITC; VCT; INDEX; COMMUNITY; OTHER
Structural	arts_init_date	ART initiation date
Structural	arts_first_date	Date first observed to start ART (may have transferred in on ART)
Structural	artstart	ART start:0: No; 1: Yes
Structural	age_artstart	Age when started ART

Structural	age_artstartcat	Age categories: <1; 1-4; 5-14; 15-19; 20-24; 25-29; 30-34; 35-39; 40-44; 45-49; 50-54; 55-59; 60+
Structural	artyear	Year when started ART
Structural	merge_origartdata	master only (1); using only (2); matched (3); missing updated (4); nonmissing conflict (5)
Structural	facility	GRRH; Katoro
Structural	ontxcurrlist	On the current treatment list<br>0: No<br>1: Yes

Supplementary Table 4. Summary of variable name, variable type, and descriptions in the original dataset.

Effort-benefit trade-off curves calculation

In constructing the effort-benefit trade-off curve, we aimed to illustrate the balance between the resources allocated to proactive patient support and the effectiveness of identifying at-risk patients. We define *Effort* as the proportion of the total population that receives proactive support. Using the predicted probabilities of a binary outcome (e.g., risk of LTFU status) for each patient, we rank patients in descending order by their predicted probabilities and select the top *M* patients with the highest risk scores. Here, *Effort* is calculated as the fraction of the population that receives support, represented by the formula *Effort* = *M* / *N*, where *M* is the number of supported patients and *N* is the total population. For each selection of *M*, we calculate the true positive rate (TPR), defined as the proportion of correctly identified at-risk patients among those *M* selected (*TPR* = *TP* / *M*, where *TP* is the count of true positives within the top *M* patients). We generate the curve by varying *M* from 1 to *N* and plotting each point where *Effort* (*M* / *N*) is represented on the x-axis and the corresponding TPR (*TP* / *M*) is shown on the y-axis.

Attention score extraction

Attention scores are extracted through a multi-step process. First, the prompt is tokenized, and autoregressive token generation is performed. In this process, tokens are generated sequentially by the model in a loop; at each step, the model uses the previous tokens and current input to predict the next token while recording attention scores at each step. These attention scores from each layer represent the model's focus on each token relative to others while generating the sequence. Next, for the summarized EMR-enhanced LLM model, we specify keywords in the summary input prompt that are associated with each predictor and identify their subsequences within the tokenized input by locating start and end indices, grouping these tokens for attention analysis. For the summarized and raw EMR-enhanced LLM model, we specify variable names in the raw EMR input prompt and identify the subsequences corresponding to these variable names. We then aggregate attention scores for each feature across layers and compute the average attention score for each feature group. The features with the highest average attention scores indicate the model's strongest focus, thereby carrying more weight in our LLM model.

Most included and most omitted variables in the LLM summarization task

To understand which predictors were most frequently included or omitted by the longitudinal predictor summarization prompt described in our methods section, we first prepare a dataset containing the textual summaries of longitudinal patient records. We then define a set of clinically relevant predictor categories (such as demographic information, laboratory test results, medication adherence, clinical visits, and historical clinical events), each associated with explicit keywords or terms. Next, using NLP methods, we automatically scanned each EMR summary for occurrences of these category-specific keywords, enabling us to quantify the frequency of each predictor category's inclusion across all summaries. We found that the top 5 most included predictors are: sex, age, lost-to-follow-up history, missed appointments, and ART adherence. The top 5 most omitted predictors are: family planning, TB, treatment plan, scheduled visit dates, facility.

This frequency analysis allows us to clearly identify predictors that the summarization prompt most consistently included, as well as those frequently omitted, reflecting differences in their perceived clinical immediacy or data update frequency within patient records.

**Internal** validation in the Kagera Region			**External** validation in the Geita Region		
Risk category	Viral suppression	LTFU	Risk category	Viral suppression	LTFU
Enhanced LLM with **summarized** CTC3 medical records					
0.72 (0.70–0.74)	0.65 (0.64–0.66)	0.75 (0.73–0.77)	0.66 (0.63–0.69)	0.62 (0.60–0.64)	0.68 (0.65–0.71)
Enhanced LLM with **summarized and raw** CTC3 medical records					
**0.75 (0.73–0.77)**	**0.67 (0.66–0.68)**	**0.77 (0.75–0.79)**	**0.67 (0.65–0.69)**	**0.64 (0.62–0.66)**	**0.72 (0.70–0.74)**
Supervised machine learning (gradient boosting)					
0.72 (0.70–0.74)	0.68 (0.67–0.69)	0.71 (0.69–0.73)	0.66 (0.63–0.69)	0.64 (0.62–0.66)	0.65 (0.62–0.68)
Zero-shot LLM with summarized CTC3 medical records					
0.54 (0.52–0.56)	0.51 (0.49–0.53)	0.54 (0.51–0.57)	0.56 (0.53–0.59)	0.51 (0.49–0.53)	0.56 (0.54–0.58)
Zero-shot LLM with summarized and raw CTC3 medical records					
0.54	0.52	0.57	0.54	0.53	0.57

(0.52–0.56)	(0.49–0.55)	(0.55–0.59)	(0.51–0.57)	(0.51–0.55)	(0.55–0.59)

Supplementary Table 5. Comparison of model performance AUC (95% CI) on internal and external validation datasets under further de-identification of patients' information. Specifically, we perturbed all date variables by randomly shifting each participant's dates (e.g., visit date, appointment date, test date, result date) by 1 to 7 days, with the same shift magnitude applied within each individual.

Handling of transferred patients. When calculating the number of days not in care, we explicitly accounted for patients who transferred between clinics. For such patients, the period of "not in care" was computed as the number of days between the last recorded visit at the original clinic and the scheduled first visit at the new clinic, rather than treating the transfer as a loss to follow-up. This approach ensures that continuity of care is appropriately represented and that patients' transfer patterns do not artificially inflate estimates of care interruption.

Subgroup definition. The "Sex" subgroup is defined by the biological sex, which has two categories: *Male* and *Female*. The "Age" subgroup is categorized by quantile cutoffs (Q1: 26, Median: 33, Q3: 42), resulting in three age subgroups: (1) Age < 26, (2) 26 ≤ Age < 42, and (3) Age ≥ 42. "The Not-in-care history" is defined by the year in which patients were not in care, specifically: 2018 not in care, 2019 not in care, 2020 not in care, 2021 not in care, and 2022 not in care.

**Internal** validation in the Kagera Region			**External** validation in the Geita Region		
Risk category	Viral suppression	LTFU	Risk category	Viral suppression	LTFU
Enhanced LLM with **summarized** CTC3 medical records					
0.287 (0.286–0.288)	0.365 (0.364–0.366)	0.178 (0.175–0.181)	0.270 (0.269–0.270)	0.363 (0.362–0.364)	0.145 (0.144–0.146)
Enhanced LLM with **summarized and raw** CTC3 medical records					
0.290 (0.288–0.292)	0.369 (0.368–0.370)	0.180 (0.179–0.181)	0.271 (0.270–0.272)	0.364 (0.363–0.365)	0.148 (0.147–0.149)
Supervised machine learning (gradient boosting)					

0.286 (0.285–0.287)	0.369 (0.368–0.370)	0.173 (0.170–0.177)	0.270 (0.269–0.270)	0.364 (0.363–0.365)	0.140 (0.139–0.141)
Zero-shot LLM with summarized CTC3 medical records					
0.270 (0.269–0.271)	0.352 (0.351–0.353)	0.159 (0.158–0.160)	0.263 (0.262–0.264)	0.355 (0.354–0.356)	0.133 (0.132–0.134)
Zero-shot LLM with summarized and raw CTC3 medical records					
0.270 (0.269–0.271)	0.353 (0.351–0.355)	0.162 (0.161–0.163)	0.260 (0.259–0.261)	0.358 (0.357–0.359)	0.133 (0.132–0.134)

Supplementary Table 6. Comparison of model performance PR AUC (95% CI) on internal and external validation datasets.

**Internal** validation in the Kagera Region			**External** validation in the Geita Region		
Risk category	Viral suppression	LTFU	Risk category	Viral suppression	LTFU
**High risk 16825 6.95%**	**Detectable 1073 0.44%**	Unlikely 216840 89.58%	**High risk 42472 6.56%**	**Detectable 7137 1.10%**	Unlikely 574770 88.83%
**Low risk 4180 1.73%**	Suppressed 220700 91.40%	**Possibly 25223 10.42%**	**Low risk 13970 2.16%**	Suppressed 557154 86.11%	**Possibly 72260 11.17%**
**Moderate risk 4218 1.74%**	Unknown 19690 8.15%		**Moderate risk 15818 2.44%**	Unknown 82739 12.79%	
No risk 216840 89.58%			No risk 574770 88.83%		

**Training data**

Risk category	Viral suppression	LTFU
**High risk**<br>**68795**<br>**7.13%**	**Detectable**<br>**6210**<br>**0.64%**	Unlikely<br>858844<br>88.99%
**Low risk**<br>**17848**<br>**1.85%**	Suppressed<br>877309<br>90.90%	**Possibly**<br>**106283**<br>**11.01%**
**Moderate risk**<br>**19640**<br>**2.03%**	Unknown<br>81608<br>8.46%	
No risk<br>858844<br>88.99%		

Supplementary Table 7. Number and prevalence of the outcome classes. The prevalence of rare-event classes is highlighted in bold.
